# A Novel 3D Model for Visualization and Tracking of Fibroblast-Guided Directional Cancer Cell Migration

**DOI:** 10.3390/biology9100328

**Published:** 2020-10-08

**Authors:** Yihe Zhang, Bingjie Jiang, Meng Huee Lee

**Affiliations:** 1 Department of Biological Sciences, Xian Jiaotong Liverpool University, 111 Ren Ai Road, Suzhou 215123, China; yihe.zhang@xjtlu.edu.cn (Y.Z.); bingjie.jiang@xjtlu.edu.cn (B.J.); 2 Faculty of Health and Life Sciences, University of Liverpool, Brownlow Hill, Liverpool L69 7TX, UK

**Keywords:** cancer, fibroblast, 3D model, cancer migration, fibroblast-guided cancer migration

## Abstract

**Simple Summary:**

Recent advances in 3D cell culture have provided new opportunities for investigating interactions between cancer cells and their surrounding stromal cells. The 3D culture platform described herein is both time efficient and economical in the study of direct cell–cell interactions. The unique design of our dumbbell model had allowed us to visualize and monitor the entire recruitment process of cancer cells by fibroblasts under an in vitro condition. Suitable for almost every cell type, our model has the potential for a wider application as it can be adapted for use in drug screening and the study of cellular factors involved in cell–cell attraction.

**Abstract:**

Stromal fibroblasts surrounding cancer cells are a major and important constituent of the tumor microenvironment not least because they contain cancer-associated fibroblasts, a unique fibroblastic cell type that promotes tumorigenicity through extracellular matrix remodeling and secretion of soluble factors that stimulate cell differentiation and invasion. Despite much progress made in understanding the molecular mechanisms that underpin fibroblast–tumor cross-talk, relatively little is known about the way the two cell types interact from a physical contact perspective. In this study, we report a novel three-dimensional dumbbell model that would allow the physical interaction between the fibroblasts and cancer cells to be visualized and monitored by microscopy. To achieve the effect, the fibroblasts and cancer cells in 50% Matrigel suspension were seeded as independent droplets in separation from each other. To allow for cell migration and interaction, a narrow passage of Matrigel causeway was constructed in between the droplets, effectively molding the gel into the shape of a dumbbell. Under time-lapse microscopy, we were able to visualize and image the entire process of fibroblast-guided cancer cell migration event, from initial vessel-like structure formation by the fibroblasts to their subsequent invasion across the causeway, attracting and trapping the cancer cells in the process. Upon prolonged culture, the entire population of fibroblasts eventually infiltrated across the passage and condensed into a spheroid-like cell mass, encapsulating the bulk of the cancer cell population within. Suitable for almost every cell type, our model has the potential for a wider application as it can be adapted for use in drug screening and the study of cellular factors involved in cell–cell attraction.

## 1. Introduction

A growing tumor contains not only malignant cancer cells but also a large repertoire of stromal cells such as fibroblasts, infiltrated immune cells, blood vessels and a meshwork of extracellular matrix (ECM) macromolecules that lend architectural and biochemical supports to the extracellular milieu [1]. Collectively known as the tumor microenvironment, the cells and accessory molecules co-operatively engage in overlapping functions to impact cancer cell growth and development [2,3,4,5]. The roles of stromal fibroblasts in tumor progression have attracted considerable attention in recent years, as emerging evidence from cell-based and clinical research suggests a prominent role for these cells in tumor survival [6,7,8,9]. In contrast to the more quiescent, spindle-shaped normal fibroblasts usually found in the connective tissues or within fibrillar ECM of the interstitium, cancer-associated fibroblasts (CAFs) are a unique population of fibroblastic cell types renowned for their pro-tumorigenic functions [6]. Predominantly situated in the vicinity of neoplastic cells, CAFs are a heterogeneous population of irreversibly activated fibroblasts that have the ability to fuel cancer cell differentiation and invasion via ECM remodeling and production of growth factors. Normal fibroblasts, if given the right extrinsic stimuli such as growth factors and mechanical stress, can potentially be activated to exhibit CAF-like features [9]. Despite our understanding of the mechanisms by which the fibroblasts and cancer cells communicate, little is known of the way they interact from a physical contact perspective and the resulting impacts on tumor migration and development [10].

To study fibroblast–cancer cell interaction, conventional two dimensional (2D) planar culture lacks proper environmental context and structural architecture of an in vivo condition and thus cannot realistically model the complex environment of a living tissue. A mouse model, on the other hand, does not allow interactions between specific cell types to be examined in detail due to the complexity and impenetrability of the tissue environment [11]. The development of 3D culture systems that better resemble the physiological conditions in which the cells reside has thus become a powerful tool in drug discovery and tissue engineering. A growing number of studies have recently been devoted to revealing how cancer cells proliferate and migrate through the 3D matrices, and by what means the fibroblasts, CAFs in particular, support these processes [12,13]. Several interesting findings in relation to fibroblast-guided tumor cell migration have demonstrated that both the fibroblasts and cancer cells exhibited an enhanced invasiveness in 3D co-culture systems, and the cancer cells appeared to always follow the fibroblasts during the invasion process by adhering to the fibroblast protrusions [10]. In summary, these evidence are indicative of the notion that, in addition to producing growth-stimulating factors, fibroblasts also interact directly with cancer cells to regulate tumor invasion [14,15,16,17]. A major challenge for this type of studies is no doubt the difficulty in visualizing the attraction event in real time, be it under an in vitro or in vivo setting. Although imaging technologies can occasionally be used for non-invasive tracking of cells in vivo, the techniques are usually complicated, and good quality images on cell–cell interaction are hard to obtain. 

In this study, we report the design and development of a physiologically relevant three-dimensional (3D) culture model that can more realistically mimic crucial features of in vivo conditions, especially in relation to fibroblast–cancer cell interactions. Tested on the fibroblastic cell line BHK-21 and four common cancer cell variants, CaKi-1, HeLa, A375 and A549, our model is amenable to modification for all cell types in the study of cell–cell and cell–ECM interactions.

## 2. Materials and Methods

### 2.1. Materials

Unless stated otherwise, all the reagents and chemicals used in this study were purchased from ThermoScientific USA. Matrigel (BD#354234) was a product of BD Biosciences, Franklin Lakes, NJ, USA, whereas lentivirus-carrying mCherry red fluorescent protein (RFP)/luciferase dual reporter proteins was purchased directly from Genomeditech^®^, Shanghai, China. All the cell lines (BHK-21, CaKi-1, HeLa, A375, A549 and Human foreskin fibroblast, HFF) used in this study were obtained from the Shanghai Cell Bank, Chinese Academy of Science (Shanghai 200031, China), where authentication was performed by short-tandem repeat (STR) profiling. 

### 2.2. Red Fluorescent Protein (RFP) Transduction and Stable Cell Line Selection

Lentivirus-containing RFP reporter genes were transduced into CaKi-1, HeLa, A375 and A549 cancer cells in Dulbecco’s modified Eagle medium (DMEM) supplemented with 8 μg/mL polybrene for a minimum of 8 h prior to blasticidin (5 μg/mL) addition to allow for stable cell selection. To obtain cells with the highest fluorescent intensity, the transductants were subjected to a second round of selection with fluorescence-activated cell sorting cytometry at the nearby Suzhou Nano-Tech and Nano-Bionics Institute (SINANO), Chinese Academy of Science.

### 2.3. 3D Fibroblast–Cancer Cell Co-Culture Model

**Figure 1:**
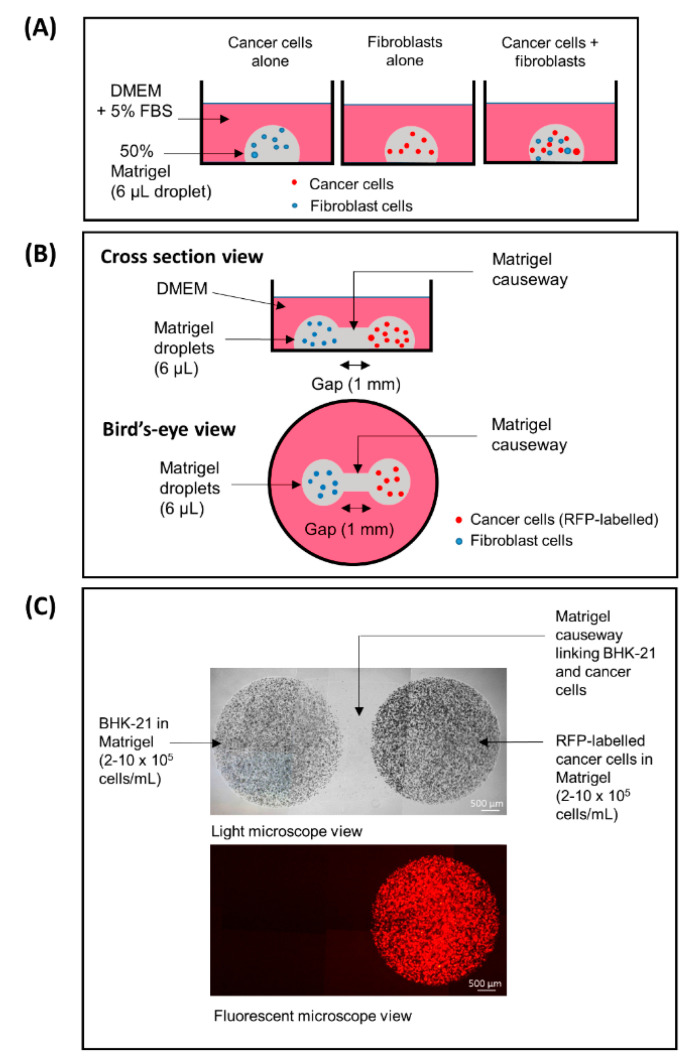
(**A**) A conventional 3D co-culture model, (**B**,**C**) a “3D dumbbell model” invented in this study for the visualization of cancer-fibroblast cell interaction. (**A**) CaKi-1 and BHK-21 fibroblast cells embedded in 50% Matrigel suspension (6 μL/droplet; containing 5 × 10^5^ cells/mL) as independent or co-culture droplets. (**B**) Caki-1 cells and BHK-21 fibroblasts in 50% Matrigel suspension (6 μL/droplet; 5 × 10^5^ cells/mL) droplets bridged by a Matrigel causeway that allows for cell migration and interaction. (**C**) Note that the cancer cells are labelled with mCherry red fluorescent protein (RFP) and thus appeared red under a fluorescent microscope. Brightfield and fluorescent views of the same cells under a 40× low power objective lens.

3D fibroblast–cancer cell co-culture model was set up essentially as illustrated in Figure 1A. Briefly, fibroblasts (BHK-21) and cancer cells (CaKi-1, HeLa, A375, A549) were premixed at the final concentration of 5 × 10^5^ cells/mL for each cell type (1:1 ratio) on ice in 50% Matrigel/DMEM supplemented with 5% fetal bovine serum (FBS). The cell–Matrigel mixture was dispensed as 6 μL micro-droplets onto the center of the wells of a 24-well tissue culture plate and allowed to solidify for at least 30 min in a 37 °C incubator prior to adding 1 mL DMEM/5% FBS. The cells were regularly inspected with a Nikon Eclipse Ti inverted microscope for morphological changes. To ensure the reproducibility of the findings, the experiments were performed in at least 12 replicates for no less than 10 times for every cancer cell type.

### 2.4. Fibroblast–Cancer 3D Dumbbell Model

Instead of co-culturing in a single droplet, the fibroblasts and cancer cells were suspended independently in separate tubes in 50% ice-cold Matrigel/DMEM at the final concentration of 5 × 10^5^ cells/mL. To create a dumbbell model, the two cell types were dispensed as 6 μL micro-droplets adjacent to each other, leaving a 1 mm gap in between the two. For the ease of explanation, a schematic representation of the model is depicted in Figure 1B,C. After the cell–Matrigel mix was incubated at 37 °C for 30 min to ensure adequate gelling, the gap between the droplets was sealed with a 5 μL “causeway” of 50% Matrigel/DMEM. The cells were monitored periodically for their moving trends by a Nikon Eclipse Ti inverted microscope or a Zeiss LSM880 Airyscan confocal microscope. To ensure the reproducibility of the findings, the experiments were typically performed in 12 replicates for a minimum of 10 times for each cancer cell type.

### 2.5. Boyden Chamber Transwell Migration Assay

For transwell migration assay, 5 × 10^4^ CaKi-1 cells pre-labelled with mCherry RFP were seeded in triplicate in 8 μm pore size Boyden chambers, while the lower compartment (typically a 24-well tissue culture plate) was filled with either the same number of BHK-21 fibroblasts or no cells (to serve as negative controls) in 1 mL DMEM/1% FBS. Incubation was allowed for 48 h at 37 °C before the membrane was transferred to a new 24-well plate and the number of migrated CaKi-1 cells counted using a Nikon Eclipse Ti inverted microscope under a 588 nm wavelength filter. Statistical significance between the control and study groups was analyzed using the Student’s T test calculator in the socscistatistics.com website.

## 3. Results

### 3.1. Spontaneous Vessel-Like Structure and Spheroid Formation by BHK-21 Cells in Matrigel Suspension

**Figure 2:**
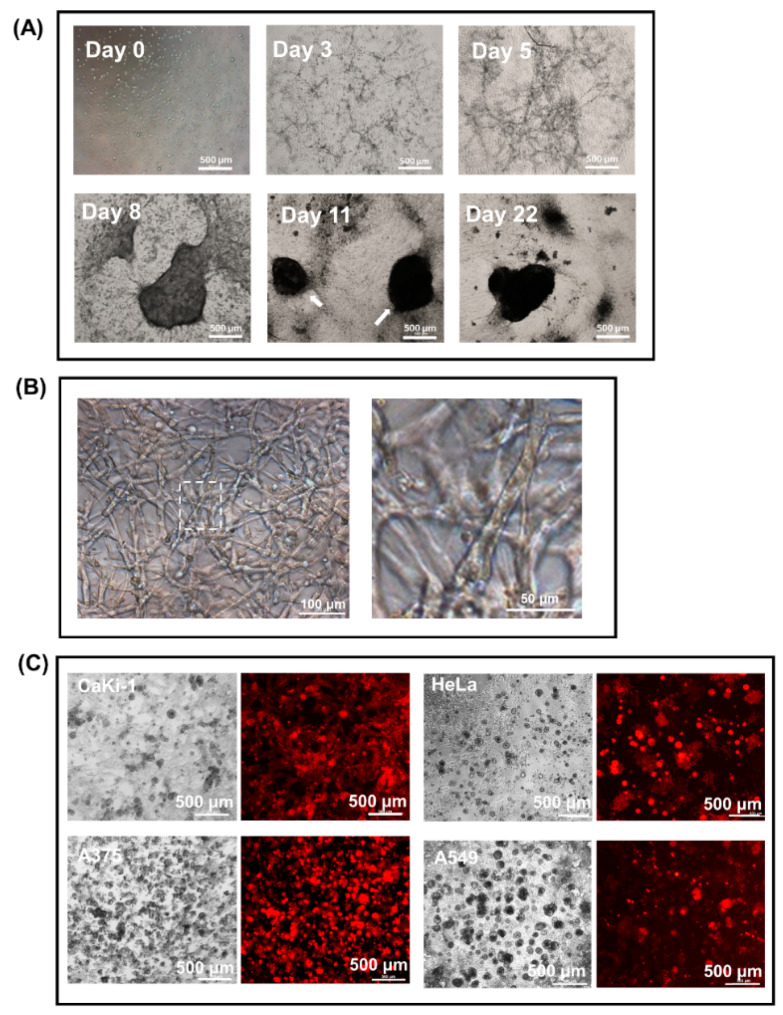
Consolidation of BHK-21 fibroblasts in Matrigel suspension into vessel-like structures and spheroid-like cell mass. (**A**) Time-lapse images showing BHK-21 vessel-like structures on days 0, 3, 5, 8, 11 and 22 that eventually morphed into a spheroid-like cell mass in a 50% Matrigel droplet. (**B**) Enlarged images of BHK-21 vessel-like structures. (**C**) Instead of developing into a network of vessel-like structures, cancer cells proliferate as small, independent colonies in 50% Matrigel suspension. Shown in the figure are CaKi-1 kidney carcinoma cells (12 days), HeLa cervical cancer cells (10 days), A375 human melanoma cells (7 days) and A549 lung adenocarcinoma cells (12 days) labelled with RFP after 7 to 12 days of culture as viewed under a fluorescent microscope.

In contrast to the 2D monolayer culture condition, BHK-21 fibroblastic cells spontaneously consolidated into a distinct network of vessel-like structures within 5 days of seeding in 50% Matrigel suspension (Figure 2A; day 5). Close examination under a 100× objective lens revealed that the vessel-like structures were made up of a network of crisscrossing and highly stretched cell bundles anywhere between 50 and 500 μm in length and one to four cells in thickness (Figure 2B; right, enlarged view). As the density and diameter of the vessel-like structures increased with time in culture, the growing contractile tension within the network caused the bulk of the cell mass to be drawn radially inward (Figure 2A, day 8) and turned into a number of compact, irregular-shaped nodules (day 11; highlighted by arrows) that, if incubation was allowed to proceed for over 20 days, would eventually morph into a single dense spheroid-like structure (Figure 2A, day 22). Under the condition stipulated in this study, the concentration of the fibroblastic cells was found not to be overly critical, as any cell number between 2 and 8 × 10^5^/mL would yield the same spheroid outcomes.

Unlike their fibroblast counterpart, cancer cells such as CaKi-1 (renal carcinoma), HeLa (cervical adenocarcinoma), A375 (melanoma) and A549 (lung adenocarcinoma) have no ability to develop into vessel-like structures in the same Matrigel suspension. Instead, they proliferated as independent colonies of varied sizes, as shown in Figure 2C.

### 3.2. Encapsulation of Cancer Cells by the Fibroblast Spheroid in 3D Matrigel Co-Culture Suspension

**Figure 3:**
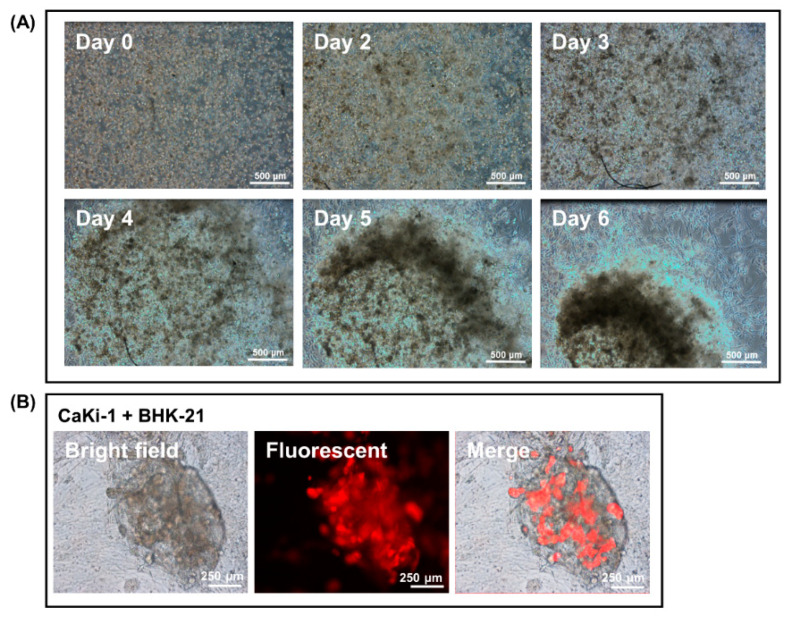
Encapsulation of cancer cells by fibroblast spheroids in a 3D Matrigel droplet. (**A**) Snapshot images from a video that show the process of CaKi-1 cell encapsulation by a fibroblast spheroid on days 0, 2, 3, 4, 5 and 6 (video in Supplement 1). (**B**) RFP-labelled CaKi-1 cancer cells trapped inside a fibroblast spheroid from a separate experiment as viewed under brightfield and fluorescent microscopy.

To investigate the effects that the fibroblasts have on cancer cell proliferation, CaKi-1 cells pre-labelled with RFP were added to the fibroblast–Matrigel mix before the cells were plated out as droplets. As in previous case, fibroblasts swiftly consolidated into a network of vessels within 5 days of seeding that, upon prolonged culture, evolved into a dense spheroid-like cell mass. The proliferation pattern of the cancer cells changed drastically in the presence of the fibroblasts. Instead of developing into individual colonies of different sizes, almost the entire population of the cancer cells were found to be encapsulated within the spheroid-like cell mass as their fibroblast counterpart underwent morphological changes in Matrigel suspension (Figure 3A and Supplementary Materials Video S1). Indeed, by the time the spheroid was fully developed on day 20, there were only a few CaKi-1 cells left dispersed outside the cell mass. Figure 3B is an image taken under high magnification that shows the encapsulation of RFP-labelled CaKi-1 cells by the fibroblasts in Matrigel suspension.

### 3.3. Adherence of Cancer Cells to Fibroblast Vessel-Like Structures in Matrigel Suspension

**Figure 4:**
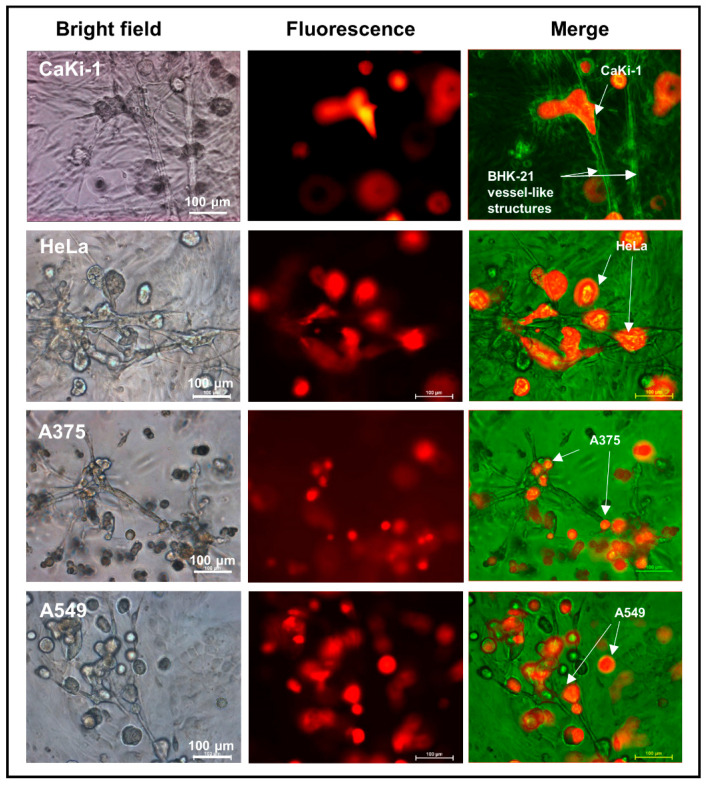
Propensity of the cancer cells to adhere to the fibroblast vessel-like structures in a 3D Matrigel suspension. Representative images showing adherence of RFP-labelled cancer cells (CaKi-1, HeLa, A375 and A549) to BHK-21 vessel-like structures in Matrigel droplets 4 days after the cells were seeded.

**Figure 5:**
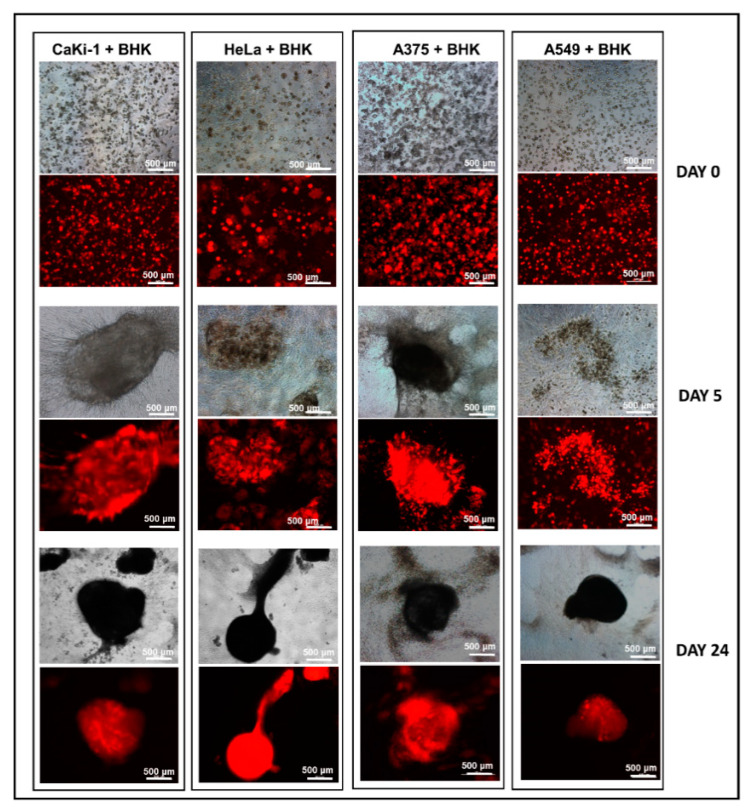
Encapsulation of cancer cells by fibroblast spheroids in 3D Matrigel. CaKi-1, HeLa, A375 and A549 were co-cultured with BHK-21 in 50% Matrigel for a period of up to 30 days. As shown by the fluorescent microscope images, almost the entire cancer cell population was encapsulated within the spheroids by the end of day 24.

To find out if the encapsulation effect was anything more complex than simple clogging of the vessel-like meshwork, we sought to monitor every step of the vessel evolvement under a high-magnification microscope. As shown in Figure 4, a vast majority of the CaKi-1 cells were found to be in adherence with the fibroblast vessels as soon as the structures began to take shape on day 4, an occurrence that well preceded the formation of the spheroid. Apart from CaKi-1, we also extended our investigation to three other cancer cell lines, namely, HeLa, A375 and A549, with similar outcomes (Figure 4). Figure 5 is a summary of snapshots that highlight the transition of the fibroblast morphology over a period of 24 days and the resulting encapsulation of the cancer cells within the spheroid-like cell mass. Apart from BHK-21 cells, similar interaction was also found in human foreskin fibroblasts (shown in Supplement 2).

### 3.4. 3D Dumbbell Model: Visualization of Fibroblast-Guided Directional Cancer Cell Migration and Encapsulation by Pioneering Fibroblast Filaments

**Figure 6:**
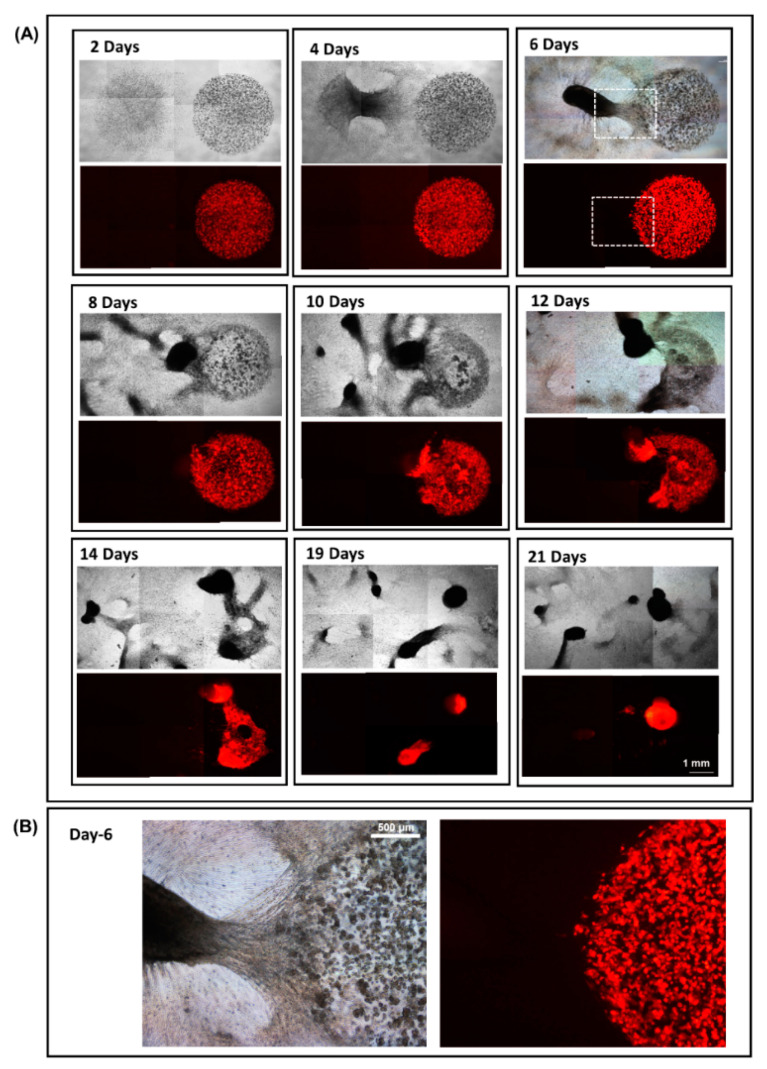
A 3D dumbbell model designed to study fibroblast-guided cancer cell migration. (**A**) Fibroblast and CaKi-1 cells were seeded as independent droplets separated by a 1 mm Matrigel causeway. Upon forming a meshwork of vessel-like structures, fibroblast cells swiftly migrated across the causeway to infiltrate and eventually encapsulate the entire CaKi-1 cell population within a spheroid. (**B**) Enlarged view of the fibroblast invasion front on day 6. Note that the cancer cells are pre-labelled with RFP.

To visualize and monitor the migration and encapsulation processes in greater clarity, we here introduced a modification to the existing model by plating the fibroblasts and cancer cells as independent droplets away from each other (shown in Figure 1B). To allow for cell migration, a Matrigel causeway was constructed in between the two droplets, effectively molding the structure into the shape of a dumbbell (Figure 1B,C). Time-lapse images show that, while the cancer cells were all proliferating as individual colonies, the fibroblasts had by the end of day 4 consolidated into a solid network of vessels within the Matrigel suspension (Figure 6A: day 2 to 4). As the vessel-like meshwork increased in number and density, the causeway linking the two droplets became an escape outlet that could offer the overcrowded fibroblast cells a passage for further expansion. Owed largely to the unique dumbbell-shaped design of the model, fibroblast vessels advancing along the narrow causeway were compressed into a compact cocoon-shaped migrating cell mass with a clear “open end” pointing towards the travelling direction (Figure 6A, day 6, highlighted in box). This “open end”, as shown by the high-magnification images in Figure 6B, is made up of a projectile of hyper-elastic pioneering fibroblast filaments. Indeed, images collected from day 4 and 6 suggested that no sooner had the fibroblasts consolidated into a vessel-like network than the cells started to migrate across the causeway, adhering and trapping in the process the surrounding CaKi-1 cells that had by now developed into small multi-cellular colonies (Figure 6B).

Incubation for a further two to four days allowed the vast majority of the fibroblasts to reach the end of the causeway and into the cancer cells’ territory (Figure 6A: day 8 to 10). Due to their inherent affinity for the fibroblast vessel-like structures, the cancer colonies progressively succumbed to infiltration and engulfment by the fibroblast cells until their eventual encapsulation inside a dense spheroid, as shown by the sequence of images taken from day 12 to 21.

Throughout the 3 weeks incubation period, we had been able to observe and monitor the entire process of cancer cell encapsulation, from the initial formation of a fibroblast vessel-like network to eventual entrapment of the CaKi-1 colonies using the dumbbell model described herein.

### 3.5. Fibroblast-Guided Cancer Cell Migration as Viewed from the Invasion Front

**Figure 7:**
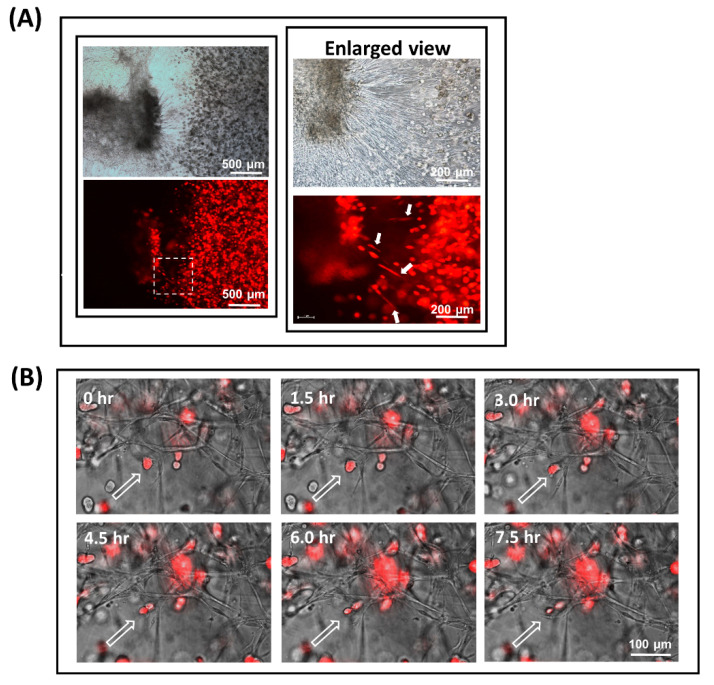
Fibroblast-guided cancer cell migration as viewed from the invasion front. Evidence demonstrating how the metastatic pathway of a cancer cell could potentially be affected by the fibroblast filaments it is in adherence to. (**A**) High-magnification images of the invasion front where fibroblasts and CaKi-1 cells first came into physical contact. Due to the elasticity and contractile motion of the pioneering filaments, some of the CaKi-1 cells in adherence to the fibroblasts appeared highly stretched and elongated (highlighted by arrows). (**B**) Time-lapse images showing a CaKi-1 cell (highlighted by an arrowhead) being coerced into a shifting position as the fibroblast filament it was attached to underwent contraction (see video in Supplementary 3).

**Figure 8:**
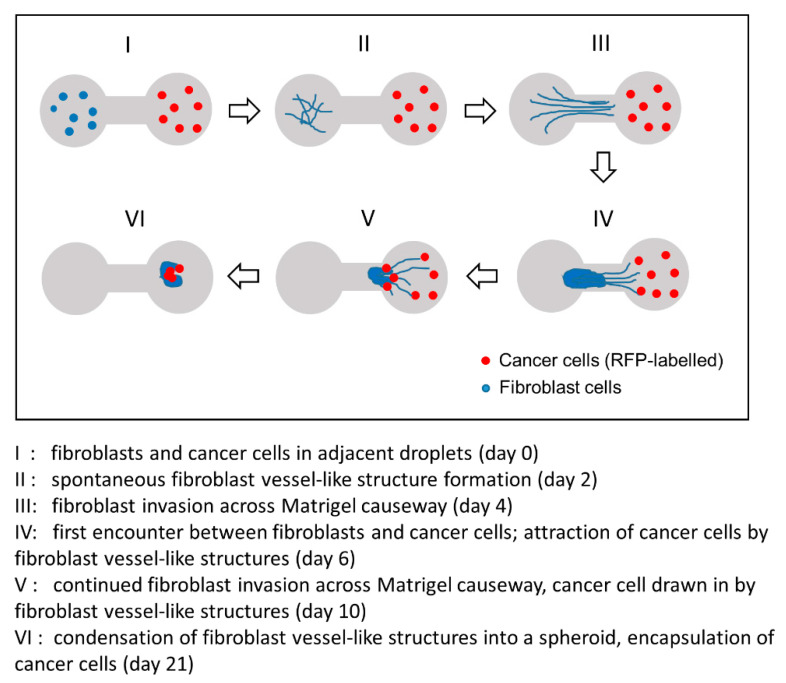
Schematic representation of a fibroblast-guided cancer cell migration event captured with our 3D dumbbell model. From initial consolidation into a vessel-like network (day 2) to eventual encapsulation of the cancer cells within a spheroid-like cell mass (day 21), our dumbbell model allows the entire invasion and fibroblast-guided cancer cell migration event to be monitored and captured in real time over a period of over 20 days.

Focusing our attention along the leading edge of the invasion front, Figure 7A (right, enlarged view) is an image that illustrates how the directionality of a cancer cell’s movement could be affected by the fibroblast vessel-like structures it is attached to. Shown in the figure are a number of highly stretched Caki-1 cells (highlighted by arrowheads) that were in firm adherence to some pioneering filaments captured in the middle of a contracting action. As supporting evidence, Figure 7B is a collage of video snapshots collected over 7.5 h that demonstrate how a RFP-labelled CaKi-1 cell (highlighted by an arrowhead) was coerced into a shifting position as the fibroblast filament it was adhered to underwent contraction (video in Supplement 3). A schematic representation of the invasion event is summarized in Figure 8.

### 3.6. Besides Physical Adherence, Fibroblasts also Attract CaKi-1 Cells by Chemoattraction

**Figure 9:**
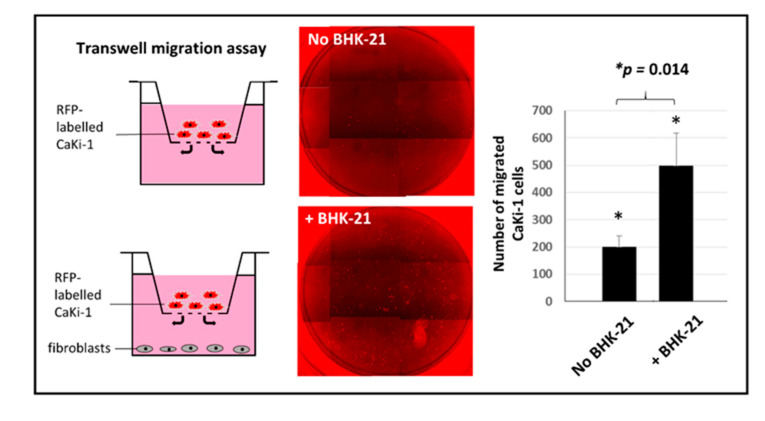
Chemoattraction of cancer cells by the fibroblasts as shown by a transwell migration assay. Addition of fibroblasts to the lower compartment of a Boyden chamber attracts significantly more CaKi-1 cells to traverse across the membrane (*p* < 0.05).

In conjunction with the dumbbell model above, we have also set up a transwell migration assay to determine whether the fibroblasts were also capable of attracting CaKi-1 cells from a distance by chemoattraction. As shown in Figure 9, culture wells filled with fibroblasts (bottom figure) attracted substantially more CaKi-1 cells to traverse across to the lower compartment than control wells with no cells (top figure) (*p* < 0.05). The results confirmed that apart from physical attraction, the fibroblasts were also capable of chemically attracting cancer cells to their vicinity, although the exact chemoattractants in this case are yet to be identified.

## 4. Discussion

The dumbbell model presented herein enables us to robustly capture the morphological changes taking place within the fibroblasts and cancer cells, including the directionality of their interactions, before and during the process of spheroid formation. To achieve the desired effect, it is imperative that the two cell types be seeded in separation to allow the fibroblasts the opportunity to first self-organize into a network of branching vessel-like structures prior to encroaching into the cancer cells’ territory. By coercing the fibroblasts into a cell mass of defined shape along a narrow causeway, we were able to observe the advancing cells as a single collective entity and, thus, gain a clear view of the invasion front, as illustrated in Figure 6. By adopting this unique design of culture platform, we were able to study the behavioral traits of the migratory cell edge and identify how the fibroblasts adapt and interact with the cancer cells as well as the surrounding matrices. In effect, the model allowed us to disentangle a somewhat complex fibroblast-directed tumor migration event into a straightforward, easy-to-follow process that would otherwise not be revealed by conventional in vivo models.

Besides visualizing cell invasion, our approach has provided us with strong experimental evidence to support the notion that cancer cells are innately attracted, physically as well as chemically, to the fibroblasts. Therefore, not only could our model provide an in vivo-like environment for the cells, it also allows the interaction dynamics between the cancer and its surrounding fibroblast cells to be visualized in a clear and unambiguous fashion. Fibroblasts, in particular CAFs, exert a physical force on cancer cells for collective invasion. More specifically, fibroblast–cancer cell co-migration requires direct cell–cell contact and adhesion possibly via the transmembrane molecules N- and E-cadherins [10,15,16,17,18]. By and large, our model allows the entire biomechanical event to be monitored and imaged in real time.

The model is simple to construct without the need for expensive reagents or specialized equipment. Furthermore, we find the results from the droplet-based platform to be robust and highly reproducible. Of note, the stiffness of the matrices was found not to be critical for both the invading and subsequent cell recruitment events, as concentration of Matrigel anywhere between 25 and 75% would yield the same outcomes. Outside the range, the cell–Matrigel mixture became either too liquidly to gel or viscous for handling. Hence, despite the numerous reports regarding the effects ECM stiffness have on the behavioral pattern of fibroblast cells [19,20], the concentration of the Matrigel does not appear to have a dramatic impact in this context. In terms of cell number, we observed no drastic difference in cell behavior so long as the number of fibroblasts and cancer cells was maintained within 2 and 10 × 10^5^ cells/mL.

## 5. Conclusions

In conclusion, the 3D dumbbell model proposed in this study is a simple and yet efficient way of studying 3D invasion and cell–cell interactions. Not only can it be used for visualization and tracking of the entire process of directed cancer cell migration and, thus, provide invaluable insights into the invasion dynamics that affect metastatic dissemination, the model is readily adaptable for other applications such as drug screening and tissue engineering.

## Supplementary Materials

The following are available online at https://www.mdpi.com/2079-7737/9/10/328/s1, Supplement 1 (video): Encapsulation of CaKi-1 cells by a fibroblast spheroid. Upon seeding in 50% Matrigel suspension, BHK-21 fibroblast cells swiftly organized into a network of vessel-like structures that subsequently morphed into a compact spheroid, carrying with them cancer cells that happened to be in the vicinity; Supplement 2: Cancer cells (CaKi-1) also exhibit a propensity to adhere to human foreskin fibroblasts (HFF) in Matrigel suspension. Albeit less intensive than their BHK-21 counterpart, HFF also form a network of loose vessel-like structures in Matrigel that are capable of attracting cancer cells. Displayed in the figure is a high-magnification (100× objective lens) image showing the adherence of CaKi-1 cells to HFF vessel-like structures in 50% Matrigel.; Supplement 3 (video): Adherence and stretching of a RFP-labelled CaKi-1 cell by the fibroblast vessel-like structures. Time-lapse images taken over 7.5 h that show how a CaKi-1 cell shifted position as the fibroblast filament it was adhered to underwent contraction.

## Patent

A patent application (202010590530.1) has been submitted to the Chinese Intellectual Property Office in June 2020 to protect the design of the dumbbell model in this report.

## Availability of Data and Materials

All the data generated or analyzed during this study are included in this published article.
